# Neutralizing Antibody Response to Influenza A(H5N1) Virus in Dairy Farm Workers, Michigan, USA

**DOI:** 10.3201/eid3104.250007

**Published:** 2025-04

**Authors:** Min Z. Levine, Feng Liu, Natasha Bagdasarian, Crystal Holiday, Stacie Jefferson, Zhu-Nan Li, Claudia Pappas, Terrence Tumpey, Timothy M. Uyeki, Alexandra M. Mellis, Krista Kniss, Joseph Coyle, Seth Eckel, Jeremy Kuo, Meghan Weinberg, Sarah Lyon-Callo, Lisa Mikesell, Becky Stoddard, Jennifer Morse

**Affiliations:** Author affiliations: Centers for Disease Control and Prevention, Atlanta, Georgia, USA (M.Z. Levine, F. Liu, C. Holiday, S. Jefferson, Z.-N. Li, C. Pappas, T. Tumpey, T.M. Uyeki, A.M. Mellis, K. Kniss); Michigan Department of Health and Human Services, Lansing, Michigan, USA (N. Bagdasarian, J. Coyle, S. Eckel, J. Kuo, M. Weinberg, S. Lyon-Callo); Mid-Michigan District Health Department, Stanton, Michigan, USA (L. Mikesell, B. Stoddard, J. Morse)

**Keywords:** Viruses, influenza, respiratory infections, highly pathogenic influenza, influenza A(H5N1), zoonoses, neutralizing antibody response, Michigan, United States

## Abstract

Since March 2024, highly pathogenic avian influenza A(H5N1) viruses have caused outbreaks in dairy cattle and poultry in the United States, and they continue to spill over into humans. However, data on human immune response to those viruses is limited. We report neutralizing antibody responses in 2 dairy farm worker H5N1 cases.

During March–December 2024, highly pathogenic avian influenza (HPAI) A(H5N1) clade 2.3.4.4b viruses caused unprecedented outbreaks in dairy cattle and poultry and spilled over into humans in the United States (*1*,*2*). By December 30, 2024, sixty-six cases of human H5N1 were reported (*3*). However, limited data are available on the immune response in those cases, including cases with only mild clinical illness, such as conjunctivitis. 

H5N1 infections have been identified in occupationally exposed persons in multiple US states, including in 2 Michigan dairy farm workers who were H5-positive by reverse transcription PCR (RT-PCR); 1 (MI-A) experienced conjunctivitis and the other (MI-B) acute respiratory illness (ARI) (*4*). We assessed antibody response in those 2 patients.

We collected paired serum samples from each worker at 2 time points after symptom onset: serum sample 1 (S1) during the acute phase (day 9 for MI-A, day 11 for MI-B) and serum sample 2 (S2) during the convalescent phase (day 31). We analyzed serum samples by microneutralization (MN) assay (*5*,*6*) and hemagglutination inhibition (HI) assay (*5*,*6*) against wild type 2.3.4.4b A/Texas/37/2024 H5N1 virus in Biosafety Level 3 enhanced (BSL-3E) laboratories (Appendix). We also analyzed neutralizing antibodies to a recently circulating group 1 seasonal influenza A(H1N1)pdm09 virus, A/Victoria/2570/2019. 

To assess antibody response to H5N1 virus infection, World Health Organization (WHO) guidelines recommend paired serum samples, paired serum samples are recommended (*7*); acute serum should be collected <7 days after symptom onset and convalescent serum at >21 days, ideally 21–28 days, after symptom onset (*7*). 

WHO defines seroconversion as a >4-fold rise in neutralizing or HI antibody titers (S2/S1) and S2 >40 (*7*) and seropositivity as both neutralizing and HI antibody titers >40 (*7*). To confirm that the detected antibody response was specific to H5N1 clade 2.3.4.4b virus infection and not from cross-reactivity with seasonal influenza viruses, we further adsorbed seropositive samples with hemagglutinins (HAs) from seasonal influenza A(H1N1)pdm09 and A(H3N2) viruses (*8*) (Appendix).

Dairy worker MI-A had conjunctivitis after milk splashed into the eye while working at a farm with confirmed HPAI H5N1 in dairy cattle (*4*). A conjunctival specimen was positive for influenza H5 by real-time RT-PCR (cycle threshold 28); H5N1 virus was isolated from the specimen (A/Michigan/90/2024) and sequenced as clade 2.3.4.4b genotype B3.13, as previously reported (*4*). The amino acid sequence of the HA1 from A/Michigan/90/2024 was 100% identical to the HA1 of A/Texas/37/2024 (*9*) (Appendix Figure). From day 9 to day 31 after symptom onset, MI-A mounted a 2.9-fold rise in neutralizing antibody (MN titers 28 to 80) and HI antibody (titers 20 to 57) responses to H5N1 clade 2.3.4.4b virus; S2 titers were >40 for both neutralizing and HI antibodies. For MI-A, S2 was seropositive to H5N1 clade 2.3.4.4b virus. However, serum samples from MI-A did not meet seroconversion criteria, likely because of the elevated baseline in S1 collected at day 9, which is after the optimal acute serum collection period (<7 days); thus, antibody response to H5N1 infection had already occurred. MI-A’s neutralizing antibodies to the seasonal influenza A(H1N1)pdm09 virus remained seronegative (MN titers 10) at both time points (Figure, panel A). Moreover, when we performed serum adsorption with seasonal influenza A(H1N1)pdm09 and A(H3N2) HAs, neutralizing antibody titers to clade 2.3.4.4b A/Texas/37/2024 H5N1 in S2 remained positive (MN titers 80), suggesting MI-A mounted antibody responses specific to H5N1 clade 2.3.4.4b virus.

**Figure:**
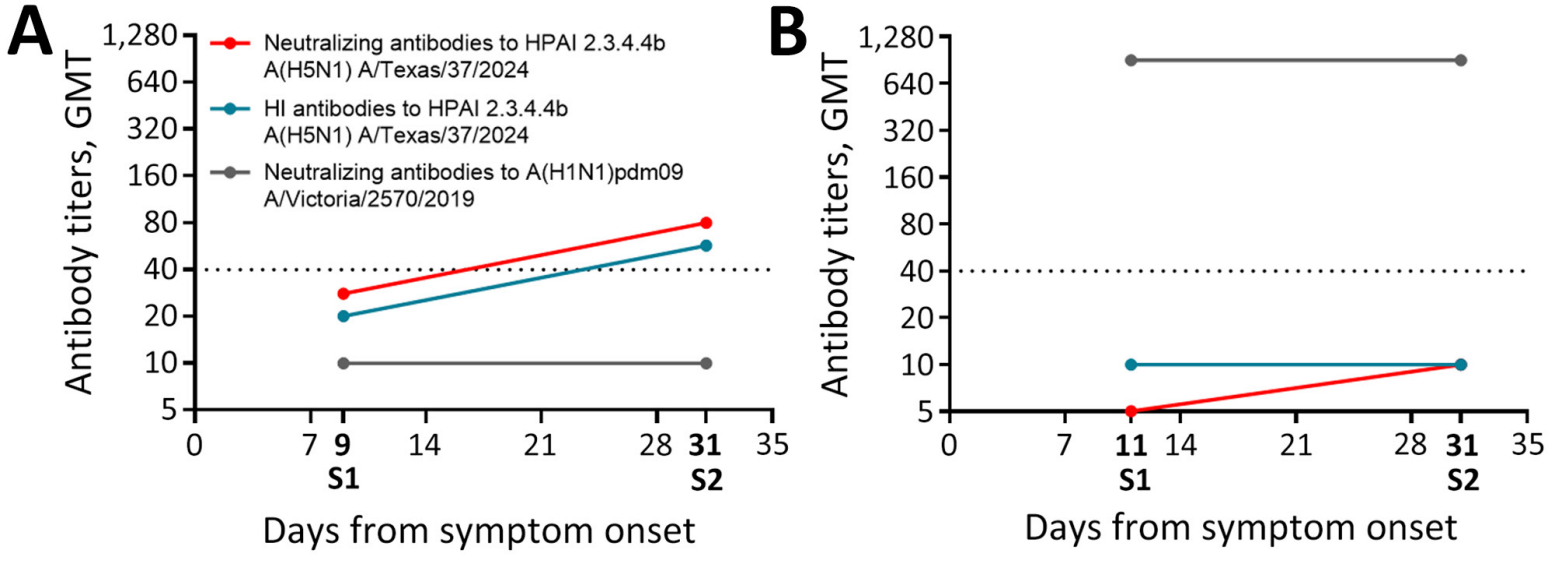
Results of microneutralization and HI assays to assess neutralizing antibody response to HPAI A(H5N1) virus infection in dairy farm workers, Michigan, USA. A) Case MI-A; B) case MI-B. We collected paired serum samples (S1 and S2) and tested against wild-type HPAI H5N1 clade 2.3.4.4b virus. Serum samples were tested at 1:10 predilution, and titer <10 was reported as 5. Dashed lines denote seropositive (titer >40) threshold at 40. GMT, geometric mean titer; HI, hemagglutination inhibition; HPAI, highly pathogenic avian influenza; S1, serum sample 1 (acute phase); S2, serum sample 2 (convalescent phase).

Dairy worker MI-B had ARI (*4*). A nasopharyngeal swab specimen was positive for influenza H5 (cycle threshold 33), but no virus was isolated, and only a partial HA sequence was obtained because of low viral load in the clinical specimen, as previously reported (*4*). Sequencing also confirmed an H5N1 clade 2.3.4.4b virus (*4*); the HA1 of the partial sequence was 100% identical to A/Michigan/90/2024 and A/Texas/37/2024 (Appendix Figure). In MI-B, we did not detect neutralizing or HI antibodies to H5N1 clade 2.3.4.4b virus (titer <10) in samples from day 11 (S1) nor 31 (S2) after symptom onset (Figure, panel B). MI-B had high and stable levels of preexisting neutralizing antibodies (titer 905) to a recent seasonal influenza A(H1N1)pdm09 virus (A/Victoria/2570/2019) at both time points (Figure, panel B), suggesting past vaccination or infection with seasonal influenza viruses.

Our results demonstrate that human HPAI H5N1 clade 2.3.4.4b virus infection, even in patients with clinically mild illness and localized infection, such as conjunctivitis, can induce neutralizing antibody responses. Unlike previously reported cases of H5N1 virus infection elsewhere that often had severe disease and high fatality rates, thus far, most human cases in the United States manifested mild clinical symptoms (*2*). Furthermore, MI-B experienced ARI but had low viral load and no virus isolation and did not have a detectable neutralizing antibody response, suggesting that not all RT-PCR–positive cases have detectable antibody responses. 

In conclusion, understanding immune response to HPAI H5N1 viruses in humans is critical. Continued efforts are needed to assess factors (e.g., viral load) that can affect antibody responses in human cases and whether preexisting immunity and immune response to infection (e.g., neutralizing antibodies and other immune responses such as neuraminidase antibodies) are associated with disease severity and clinical outcomes. Continued serosurveillance will be crucial for assessing pandemic risk of influenza A(H5) viruses.

Appendix Additional information on neutralizing antibody response to HPAI A(H5N1) virus infection in dairy farm workers, Michigan, USA.
