# Supplementary material for: Neutralizing Antibody Response to Influenza A(H5N1) Virus in Dairy Farm Workers, Michigan, USA
Source: Emerg Infect Dis. 2025 Apr;31(4):876–8. doi: 10.3201/eid3104.250007 (PMC11950262; doi:10.3201/eid3104.250007)
Supplement: Appendix — Additional information on neutralizing antibody response to HPAI A(H5N1) virus infection in dairy farm workers, Michigan, USA. [file 25-0007-Techapp-s1.pdf]

Article DOI: https://doi.org/10.3201/eid3104.250007

EID cannot ensure accessibility for supplementary materials supplied by authors. Readers who have difficulty accessing supplementary content should contact the authors for assistance.

Neutralizing Antibody Response to Influenza A(H5N1) Infection in Dairy Farm Workers, Michigan, USA

Appendix

Laboratory Methods

Hemagglutination Inhibition Assay

Hemagglutination inhibition (HI) assays to detect antibody responses to A(H5N1) viruses were performed with a modified HI assay using horse erythrocytes optimized for avian influenza H5 viruses as previously described (*1*,*2*). In brief, sera were heat inactivated for 30 minutes at 56°C and then tested for non-specific agglutinins and adsorbed with horse erythrocytes, if needed. Sera were then treated with receptor-destroying enzyme for 18–20 hours at 37°C to remove any non-specific inhibitors that may have been introduced during hemadsorption, followed by heat inactivation before the HI assay. Sera were first pre-diluted at 1:10, then serially diluted 2-fold and incubated for 30 minutes with 4 hemagglutination units per 25 µL of virus, the serum-virus mixture was then incubated with 1% horse erythrocytes for 60 minutes. HI antibody titer was defined as the reciprocal of the last dilution of serum that completely inhibited hemagglutination. Antibody titer <10 (initial dilution) was reported as 5 for calculation purpose. Multiple replicates were conducted, reported titers are geometric mean titers (GMTs) from multiple replicates.

HI assays were conducted at Centers for Disease Control and prevention (CDC) in Biosafety Level 3 enhanced (BSL-3E) laboratories.

Microneutralization Assay (MN)

Microneutralization (MN) assays were performed as previously described (*1*,*2*). Heat inactivated human sera were pre-diluted at 1:10, then serially diluted 2-fold and incubated with

100 50% tissue culture infection dose ($TCID_{50}$) of influenza viruses. The virus-sera mixture was used to infect 1.5×10^4/well Madin-Darby canine kidney (MDCK) cells and incubated overnight. The plates were fixed with cold 80% acetone and the presence of viral nucleoprotein was quantified by enzyme-linked immunosorbent assay (ELISA). Neutralizing antibody titers were defined as the reciprocal of the highest serum dilution that showed 50% neutralization. Antibody titer <10 (initial dilution) was reported as 5. Multiple replicates were conducted, reported titers are geometric mean titers (GMTs) from multiple replicates.

MN assays were conducted in CDC BSL-3E laboratories.

Sequence analysis

Hemagglutinin (HA) sequences of A/Michigan/90/2024 [Global Initiative on Sharing Avian Influenza Data (GISAID) EPI3334182], A/Michigan/91/2024 (EPI3352530) and A/Texas/37/2024 [GISAID EPI3171488] were analyzed by Bioedit version 7.0.9.0.

Serum adsorption

Both dairy farm workers had reverse transcription–polymerase-chain-reaction (RT-PCR) -confirmed influenza (AH5N1) virus infections at the time of acute serum collection. MI-A had evidence of a rise in neutralizing antibody titers to A(H5N1) virus but not to A(H1N1)pdm09 virus post-infection, suggesting detection of a specific antibody response to A(H5N1) 2.3.4.4b virus. To further confirm that the antibody response detected in MI-A's convalescent serum was specific to 2.3.4.4b A(H5N1) virus infection, and not from cross-reactivity with seasonal influenza A viruses, the convalescent serum (S2) from MI-A which was determined to be seropositive to A(H5N1), was further adsorbed with hemagglutinin (HA) from seasonal A(H1N1)pdm09 and A(H3N2) influenza viruses using methods previously described (*3*). Recombinant his-tagged HA1 (rHA1) from recent circulating seasonal A(H1N1)pdm09 (A/Wisconsin/588/2019) and A(H3N2) (A/Darwin/6/2021) viruses were conjugated with nickel magnetic beads. The beads were washed with phosphate-buffered saline (PBS) to remove unbound rHA1. Then serum was incubated and adsorbed with A(H1N1)pdm09 and A(H3N2) rHA1 conjugated beads at room temperature for 60 min to remove any potential cross-reactive antibodies. The beads were then removed by centrifugation. Post-adsorption MI-A convalescent serum (S2) was then tested again in MN assays against 2.3.4.4b A/Texas/37/2024 H5N1 and seasonal A/Victoria/2570 H1N1 pdm09 viruses.

References

1. Levine MZ, Holiday C, Liu F, Jefferson S, Gillis E, Bellamy AR, et al. Cross-Reactive Antibody Responses to Novel H5Nx Influenza Viruses Following Homologous and Heterologous Prime-Boost Vaccination with a Prepandemic Stockpiled A(H5N1) Vaccine in Humans. J Infect Dis. 2017;216(suppl_4):S555–9. PubMed https://doi.org/10.1093/infdis/jix001

2. Levine MZ, Holiday C, Jefferson S, Gross FL, Liu F, Li S, et al. Heterologous prime-boost with A(H5N1) pandemic influenza vaccines induces broader cross-clade antibody responses than homologous prime-boost. NPJ Vaccines. 2019;4:22. PubMed https://doi.org/10.1038/s41541-019-0114-8

3. Li ZN, Cheng E, Poirot E, Weber KM, Carney P, Chang J, et al. Identification of novel influenza A virus exposures by an improved high-throughput multiplex MAGPIX platform and serum adsorption. Influenza Other Respir Viruses. 2020;14:129–41. PubMed https://doi.org/0.1111/irv.12695

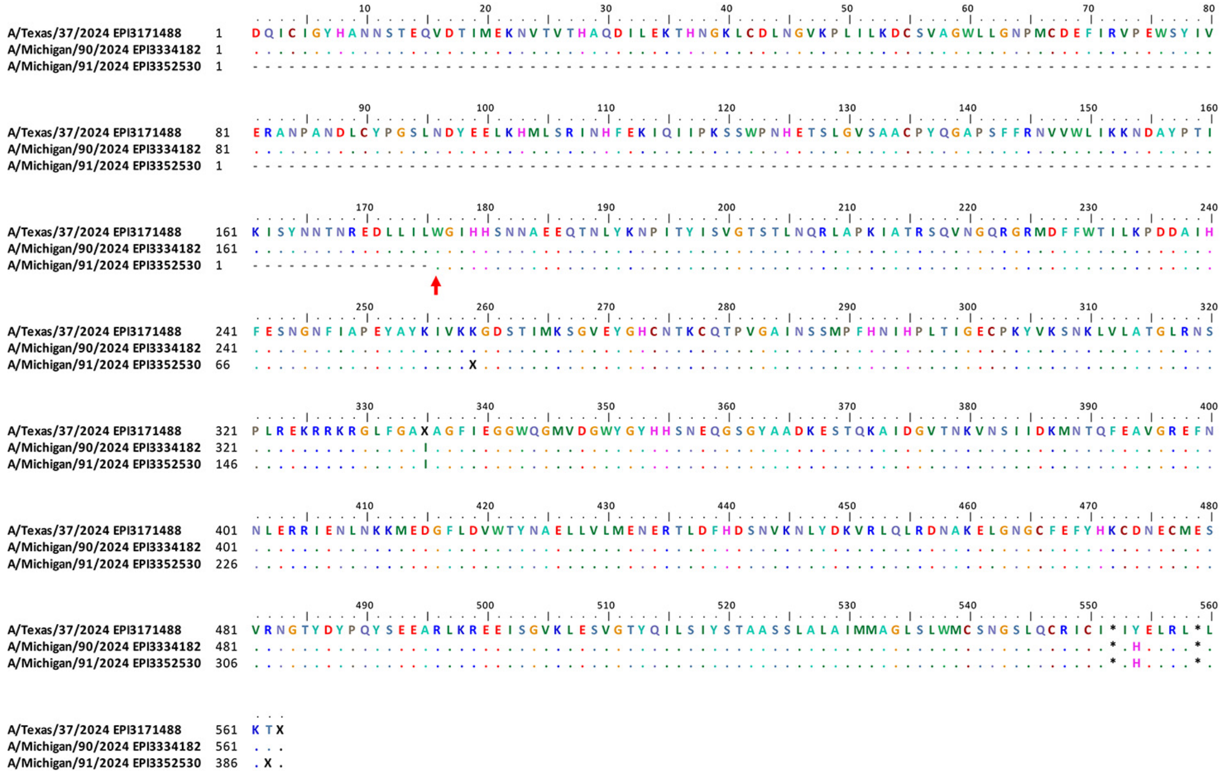

Appendix Figure. Hemagglutinin (HA) amino acid sequence alignment of 2.3.4.4b A(H5N1) viruses. The HA amino acid sequences from A/Texas/37/2024 (EPI3171488), A/Michigan/90/2024 (EPI3334182, from

MI-A), and A/Michigan/91/2024 (EPI3352530, from MI-B) A(H5N1) viruses were analyzed using ClustalW (http://www.clustal.org/clustal2) multiple alignment with BioEdit (https://bioedit.software.informer.com). Identical amino acids referenced to the HA sequence of A/Texas/37/2024 virus were presented as dots. Only partial HA sequence is available from A/Michigan/91/2024 from MI-B, started at amino acid position 176 (denoted by a red arrow).
